# The effectiveness of melatonin for promoting healthy sleep: a rapid evidence assessment of the literature

**DOI:** 10.1186/1475-2891-13-106

**Published:** 2014-11-07

**Authors:** Rebecca B Costello, Cynthia V Lentino, Courtney C Boyd, Meghan L O’Connell, Cindy C Crawford, Meredith L Sprengel, Patricia A Deuster

**Affiliations:** Department of Military and Emergency Medicine, Uniformed Services University of the Health Sciences, 4301 Jones Bridge Road, Bethesda, MD 20814-4799 USA; Office of Dietary Supplements, National Institutes of Health, 6100 Executive Blvd., Room 3B01, MSC 7517, Bethesda, MD 20892-7517 USA; Samueli Institute, 1737 King Street, Suite 600, Alexandria, VA 22314 USA

**Keywords:** Melatonin, Sleep, Systematic review, Rapid evidence assessment of the literature (REAL), Dietary supplements, Military health

## Abstract

A systematic review was conducted using Samueli Institute’s Rapid Evidence Assessment of the Literature (REAL^©^) process to determine the evidence base for melatonin as an agent to optimize sleep or improve sleep quality, and generalize the results to a military, civilian, or other healthy, active, adult population. Multiple databases were searched yielding 35 randomized controlled trials (RCTs) meeting the review’s inclusion criteria, which were assessed for methodological quality as well as for melatonin effectiveness. The majority of included studies were high quality (83.0%). Overall, according to Grading Recommendations, Assessment Development and Evaluation (GRADE) methodology, weak recommendations were made for preventing phase shifts from jet lag, for improving insomnia in both healthy volunteers and individuals with a history of insomnia, and for initiating sleep and/or improving sleep efficacy. Based on the literature to date, no recommendations for use in shift workers or to improve hormonal phase shift changes in healthy people can be made at this time. Larger and longer-duration RCTs utilizing well characterized products are needed to warrant melatonin recommendations in young, healthy adults.

## Introduction

Sleep quality is a problem in the civilian population, where 17.4% of respondents to the 2002 Alternative Health/Complementary and Alternative Medicine supplement to the National Health Interview Survey (NHIS) reported insomnia or trouble sleeping in the past 12 months [1]. Likewise, sleep quality is a concern within the military. Data are just beginning to be published regarding sleep habits and behaviors in the military. A cross-sectional study of 156 deployed US Air Force Airmen found that 40.0% of respondents suffered from at least one sleep disturbance and 75.0% reported diminished sleep quality while deployed, as compared to sleep quality at home [2]. A 2010 paper on the Millennium Cohort - 41,225 active duty and retired Service Members - demonstrated that deployment significantly compromised sleep quality and quantity [3]. Finally, in 2013, Lentino et al. [4] noted that over 25% of 14,148 Army Active, Reserve, and National Guard members reported to be “poor” sleepers, with only 32.9% self-reporting as “good” sleepers. Clearly, obtaining adequate and good quality sleep are continual challenges for active duty Service Members during training and during periods of deployment. Although sleep promoting medications, such as zolpidem, are available and widely used by military personnel for both sleep problems and fatigue management [5], they have been associated with various adverse effects, including cognitive impairments [6, 7] and in some cases “somnambulism-like state with sleep-related complex behavior.” Thus, more natural solutions to the military “sleep problem” must be sought. The dietary supplement, melatonin, may be one natural solution.

Exogenous melatonin, as both prescription and over the counter pills/tablets, has become one of the most frequently requested non-prescription sleep aids due to its regulator role in the internal timing of biological rhythms, including promotion/regulation of sleep [8]. Melatonin is marketed to help promote total sleep time, aid with fatigue from jet lag, or balance circadian rhythms from jet lag and rotating shift work. Evidence suggests melatonin may reduce the time it takes for people with delayed sleep phase syndrome (i.e., sleep is delayed by two or more hours beyond the conventional bedtime, causing difficulty in waking at a desired time) to fall asleep [9]; melatonin may also help re-set the body’s sleep-wake cycle [10]. Importantly, melatonin has been shown to serve as a mediator between the thermoregulatory and arousal system in humans, such that exogenous administration of melatonin during the day can result in sleepiness in association with reduced core temperature [11, 12].

Of interest, approximately 5.2% of the NHIS survey respondents reported using melatonin and 27.5% of those users reported insomnia as a reason for taking the supplement [1]. Some studies have shown that supplemental melatonin can increase sleep propensity, although it may not be as effective as prescription sleep medications [13]. If melatonin were found to be effective for promoting healthy sleep, it could be a useful and suitable sleep aid for military as well as other populations, where sleep problems are a serious concern.

Although a small number of systematic reviews have investigated melatonin’s effects on specific clinical conditions, to date no systematic reviews have explored melatonin across all aspects of sleep behavior in an otherwise healthy population. Thus, the purpose of this systematic review was to: 1) determine the evidence base for melatonin to optimize sleep (e.g., improve sleep quality, duration, ability to fall asleep); 2) evaluate the safety of melatonin use; 3) assess outcomes and dosing structure most applicable for this research; and 4) generalize results to those (e.g., at risk military populations) who suffer from issues related to sleep disturbances and sleep hygiene [14–21]. This review also discusses strengths, weaknesses, and gaps emerging from the review, as well as recommendations for moving this research field forward, in particular with regard to the usefulness of melatonin for military populations in need of sleep regulation as well as other populations with similar sleep issues.

## Methods

A systematic review was conducted using the Rapid Evidence Assessment of the Literature (REAL©) process, a methodology developed by Samueli Institute to provide a “snapshot” of the peer-reviewed literature in a streamlined and efficient fashion. The REAL process differs from traditional systematic reviews in that it does not “exhaustively” search the literature by including grey and non-English language literature, but instead includes only randomized controlled trial (RCT) and systematic review study designs accessible in current English electronic databases. Following REAL methodology, a research question was developed with subject matter experts (SMEs) by using the evidence-based Population, Intervention, Comparison, Outcome (PICO) framework [22] to assess the effectiveness of melatonin on mitigating poor sleep and/or promoting healthy sleep as published and reported in RCTs.

### Data sources and search strategy

PubMed/MEDLINE, CINAHL, Embase, PsycInfo, and Agricola databases were searched from database inception until October 2012 for RCTs investigating the relationship between melatonin and healthy sleep behaviors. Authors explored MeSH terminology within MEDLINE and consulted with three subject matter experts (PAD, CVL, RBC) to not only strategize the most powerful search, but to also ensure the correct key terms were being targeted for the research question proposed. The following search strategy was conducted in PubMed, and MeSH was applied where applicable: “(melatonin) and (sleep or fatigue or sleep disorders, circadian rhythm or insomnia).” Following traditional REAL methodology, which includes the assessment of RCTs involving humans and published in the English language, all searches were conducted using these parameters. Where this was not a limit option in certain databases, citations were screened for these criteria.

### Study selection

Articles were included if they met the following criteria: 1) RCT presented in the English language and involving adult human subjects; 2) healthy non-military or military populations, or populations diagnosed with insomnia, as reported by the study’s authors; 3) use of melatonin as the sole intervention; and 4) at least one sleep outcome of interest (e.g., sleep quality, sleep latency, sleep duration).

Articles were excluded if they met at least one of the following criteria: 1) any study design other than a RCT; 2) population with pre-existing conditions or diseases other than insomnia; 3) focus of article was on an intervention other than melatonin; 4) intervention was a combination of melatonin and other supplements or drugs; or 5) article did not have at least one sleep outcome of interest.

### Data extraction

In order to streamline the systematic review process in a secure manner and ensure reliability and consistency across reviewer ratings, the authors conducted the review within the web-based systematic review management program Mobius Analytics SRS (Copyright 2003–2009 Mobius Analytics Inc, Ottawa, Ontario). This program reduces errors and post-review data collation, and increases reviewer efficiency by automating article progression and management.

Articles meeting the inclusion criteria were assessed for methodological quality using the Scottish Intercollegiate Guidelines Network (SIGN 50) checklist, a reliable and valid assessment tool widely used in the literature [23] (Table 1). Three trained reviewers (MLO, MLS, CCB) reviewed articles in pairs until a sufficient kappa (>90%) was achieved, at which point they independently reviewed the remaining articles. All work was cross-checked by the review manager (CCC), and disagreements were resolved either through discussion and consensus, or by one of the SMEs.

**Table 1:** **Sign 50 checklist for RCT study design** [23]

**Section 1: Internal validity** ^**1**^	
**Item**	**Criteria**
1.1	The study addresses an appropriate and clearly focused question.
1.2	The assignment of subjects to treatment groups is randomized.
1.3	An adequate concealment method is used.
1.4	Subjects and investigators are kept blind about treatment allocation.
1.5	The treatment and control groups are similar at the start of the trial.
1.6	The only difference between groups is the treatment under investigation.
1.7	All relevant outcomes are measured in a standard, valid and reliable way.
1.8	What percentage of subjects in each treatment arm dropped out before the study was completed?
1.9	All subjects are analyzed in the groups to which they were randomly allocated (intention-treat analysis).
1.10	Where the study is carried out at more than one site, results are comparable for all sites.
**Section 2. Overall Assessment**	
**Quality Score**	**Criteria**
**++**	**All or most** of the criteria have been fulfilled adequately or well. Where they have not been fulfilled the conclusions of the study are thought *very unlikely* to alter. *An article receives this score if there are 0 criteria scored as poorly addressed.*
**+**	**Some** of the criteria have been fulfilled adequately or well. Those criteria that have not been fulfilled or not adequately described are thought *unlikely* to alter the conclusions. *An article receives this score if 1-3 criteria are scored as poorly addressed.*
-	**Few or no** criteria fulfilled adequately or well (3 or more poorly addressed criteria). The conclusions of the study are thought *likely or very likely* to alter. *An article receives this score if more than 3 criteria are scored as poorly addressed.*

^1^Each item is evaluated as well covered, adequately addressed or poorly addressed. Item 1.10 can also be marked as not applicable.

The following descriptive data were extracted for each of the included studies: population description, sample size, melatonin and control interventions and dosages, all sleep related outcomes and statistics, funding source, author’s main conclusions and whether power calculations, adverse events, and cost analyses were reported. Additional study design elements deemed important for quality control of dietary supplement studies were also extracted from each study, including characterization of the intervention product, baseline exposure or background diet (e.g. use of dietary supplements) of the study participants, and whether or not dietary intake was controlled during the study. Additionally, data regarding melatonin supplement formulation and whether or not it was analyzed for purity and absorption after ingestion were gathered.

### Data synthesis and analysis

Once the quality assessment of individual RCT study reports was completed, the SMEs performed a quality assessment of the overall literature pool for each identified population by using a modification of the Grading of Recommendation Assessment, Development and Evaluation (GRADE) [24], an internationally accepted approach to grading the quality of evidence and strength of recommendations across studies. SMEs were trained in this methodology and utilized a grading rulebook developed, tested and agreed upon by the entire team. SMEs examined the outcomes of the individual RCTs for each category of intended use (i.e., shift workers, jet lag, insomnia, healthy volunteers) in order to: 1) examine the confidence in the estimate of the effect; 2) determine the magnitude of the effect size overall; 3) assign a safety grade to the literature; and 4) develop recommendations for the melatonin literature based on the REAL results for the overall literature pool of studies for each category. SMEs performed the GRADE analysis independently before discussing their answers together and coming to consensus with the full team. Due to the heterogeneity of included individual studies, outcome data were not pooled for statistical analysis.

## Results

**Figure 1:**
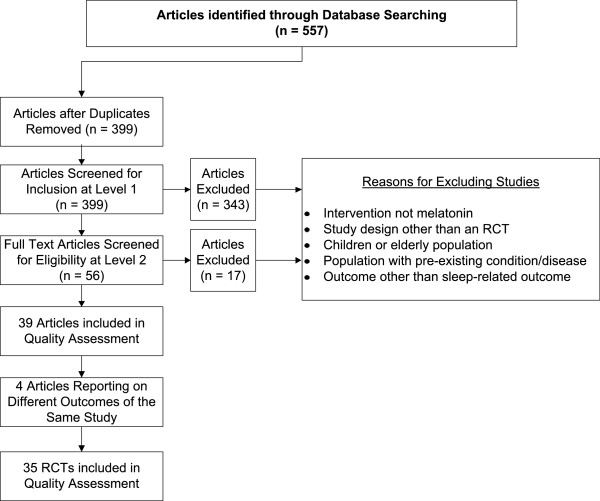
**Flow chart of included studies.**

The results of the initial database search yielded 557 references, 39 of which met the inclusion criteria and were subsequently included in the review. Articles were excluded mostly because the intervention was not melatonin or because they did not report on sleep related outcomes. Of these 39 included articles, four [25–28] reported on different outcomes of the same study and were, therefore, combined with their appropriate counterpart. Ultimately, 35 RCTs, with a total of 2,356 subjects, were included in this review (see Figure 1 for the flow chart of included studies).

Table 2 describes the characteristics of the individual studies (grouped by shift workers, jet lag, insomnia, and healthy volunteers) and overall SIGN 50 score. Table 3 describes the GRADE analysis results of the overall literature pool, and Table 4 illustrates the key design issues considered important for dietary interventional studies. Table 5 presents the number and types of subjective and/or objective assessment tools utilized in this review.

**Table 2:** **Characteristics and quality score of included studies**

Citation	Population description	Sample entered/completed	Melatonin supplement vs. control	Outcomes	Author's main conclusions	Quality ^f^
***Shift Workers (n = 8)***						
Jorgensen et al. [29]	20 emergency medicine resident and attending physicians (age/gender = ND) at the University of Maryland^ad^	overall (20/20)	10 mg melatonin pill vs. placebo taken the morning after each night shift (starting day 2)^e^	Sleep diary, SSS, sleep VAS	Melatonin did not significantly improve night alertness or day sleep in shift workers, although there was a trend toward improved night alertness.	+
James et al. [30]	24 adult emergency medical technicians or paramedics (age/gender = ND) working night shifts^ac^	overall (24/22)	6 mg melatonin pill vs. placebo capsule taken 30 min before each day sleep	Sleep diaries, sleep VAS	Melatonin supplements did not improve sleep quality or duration in emergency medical services personnel working rotating night shifts.	+
Sadeghniiat-Haghighi et al. [31]	118 healthy non-smoking non-pregnant shift-worker female nurses (age = ND) with insomnia^c^	overall (118/86)	5 mg melatonin tablet vs. placebo taken on the first night after shift work, 30 min before habitual nighttime sleep^e^	Questionnaire	Melatonin significantly decreased sleep onset latency (p = NR) and increased sleep quality as compared with placebo (p < 0.05).	-
Bjorvatn et al. [32]	38 oil rig workers, age/gender ND^c^	overall (38/17)	3 mg melatonin capsule vs. placebo taken 1 h before bedtime vs. bright light (10,000 lux) applied for 30 min/day, ranging from midnight (0000) to (0500) during the night shift and from midday (1200) to (1430) during the day shift^e^	KSS, ATS, sleep diary, 5-min reaction test, actigraph	Melatonin reduced sleepiness at work during the dayshift week (p = 0.016) and subjectively increased sleep by 15-20 min per day (p = 0.05) compared to placebo. Objectives measures indicated that reaction times did not differ between conditions whereas bright light improved sleep to a minor degree (p = 0.04).	-
Cavallo et al. [33]	45 second year pediatric residents working two night float periods (16 M/29 F) with a mean age of 28.6 ± 9^ac^	overall (45/28)	3 mg melatonin fast release capsule vs. placebo taken every morning after night shift work^e^	Sleep diary, VAS, POMS	Melatonin treatment did not improve sleep duration, vigor, or fatigue in shift workers.	+
Wright et al. [34]	15 faculty emergency physicians (12 M/3 F) with a mean age of 38.6 ± ND^ac^	overall (15/15)	2 x 2.5 mg melatonin tablets vs. placebo given 30 min before bedtime	KSS, tiredness VAS, sleep VAS, drug tolerability VAS, questionnaire	Melatonin showed no benefit in a group of emergency physicians after night-shift work.	++
Sharkey et al. [35]	21 healthy adults (12 M/9 F) with a mean age of 27.0 ± 5.0^c^	overall (21/21)	1.8 mg melatonin sustained-release tablet vs. placebo taken 30 min before 2 daytime sleep episodes^e^	PSG, saliva samples, MSLT, SSS, sleep VAS, sleep diary, actigraph	Melatonin prevented the decrease in sleep time that occurs from sleeping at the ‘wrong' circadian phase (p < 0.05). Subjects taking melatonin were sleepier at bedtime (p = 0.003) on sleep day 1 compared to placebo.	+
Jockovich et al. [36]	19 volunteer emergency medicine residents (15 M/4 F) with a mean age of 28.2 ± ND^ac^	overall (19/19)	1 mg melatonin caplet vs. placebo taken 30-60 min prior to anticipated daytime sleep session following a night shift^e^	SSS, wrist actigraph	Melatonin did not improve daytime sleep for emergency physicians working night shifts.	+
***Jet Lag (n = 8)***						
Arendt et al. [37]	17 healthy volunteers (7 M/10 F) with mean age of 48.5 ± 2.2^c^	overall (17/17), melatonin (8/8), placebo (9/9)	5 mg melatonin capsule vs. placebo taken at 1800 h on the day of their transcontinental flight departure for the two preceding days, and between 2200-2400 h on the first four days after their return flight	VAS mood, VAS sleep, VAS jet lag, urine samples	Melatonin is effective in subjectively alleviating jet lag (p < 0.01) following eastward travel over eight time zones.	+
Spitzer et al. [38]	339 Norwegian physicians (203 M/136 F) with a mean age of 44 ± 7^ac^	overall (339/257)	5 mg or 0.5 mg melatonin capsules vs. placebo taken daily at bedtime on travel day and post-travel days 1-5	Columbia jet lag scale	Melatonin did not effectively treat jet lag.	-
Claustrat et al. [39]	37 participants accustomed to intercontinental flights who usually experience subsequent discomfort after an eastward journey (18 M/12 F) with a mean age of 36.3 ± 8.9 in the melatonin group and 35.7 ± 6.4 in the placebo group^d^	overall (37/27)	8 mg melatonin capsule vs. placebo taken on day 1 (2200 h) and days 2-4 at bedtime	Global treatment efficiency VAS, sleepiness and mood VAS, sleep VAS	Melatonin demonstrated an overall efficiency in alleviating jet lag (p < 0.058) in subjects who experienced significant discomfort after an eastward flight, compared to placebo.	+
Beaumont et al. [40]	27 participants from a US Air Force Reserve Unit (18 M/9 F) with a mean age of 35.3 ± 8.1^c^	overall (27/27), slow-release caffeine (9/9), melatonin (9/9), placebo (9/9)	5 mg melatonin pill vs. 300 mg slow-release caffeine vs. placebo administered preflight (1700 h) and daily from day 1 (arrival day; 1600 h) - day 5 (2300 h)	PSG, sleep diary, MSLT, piezoelectric accelerometer, sleep VAS	Melatonin decreased sleepiness subjectively (p < 0.05), but not objectively, and improved recovery sleep (p < 0.05), indicating some value for alleviating symptoms related to jet lag combined with sleep deprivation.	+
Petrie et al. [41]	20 volunteers with experience of transcontinental flights through at least 5 time zones (12 M/8 F) with an age range from 28-68^c^	overall (20/20)	5 mg melatonin capsule vs. placebo taken once a day on pre-flight days 1-3 (between 1000 h and 1200 h), during flight, and once a day for post-flight days 1-3 (between 2200-2400 h)^e^	VAS, POMS, hours of sleep, retrospective jet lag ratings	Melatonin use resulted in significantly less overall jet lag compared to placebo (p < 0.01). Subjects taking melatonin reported that they were less tired during the day and required less time to establish a normal sleeping pattern (p < 0.05) and reach their normal level of energy (p < 0.05).	+
Suhner et al. [42]	160 recruited volunteer travelers (age/gender = ND) planning a trip from Switzerland to the American continent through 6 to 9 time zones and staying there at least 1 wk. before returning^c^	melatonin (ND/35) zolpidem (ND/34) melatonin + zolpidem (ND/29)	5 mg melatonin capsule vs. placebo vs. 10 mg Zolpidem vs. a combination of 5 mg melatonin + 10 mg Zolpidem taken on the return flight (eastbound) between 1700-2100 h and during 4 consecutive days post-flight at bedtime	Sleep diary, POMS, jet lag VAS, symptom assessments, actigraph	Melatonin reduced jet lag severity to some extent (p < 0.05). However, Zolpidem 10 mg was the most effective treatment in that it significantly improved subjective sleep quality on night flights (p < 0.05), reduced over-all jet lag feelings and alleviated sleep disturbances and confusion associated with jet lag (p < 0.05).	+
Suhner et al. [43]	320 volunteers who had flights over 6-8 time zones (172 M/148 F) with a mean age of 36 ± ND^c^	overall (320/234) melatonin (ND/174) placebo (ND/60)	5 mg fast-release (FR), 0.5 mg FR, or 2 mg controlled-release melatonin vs. placebo taken once daily at bedtime during 4 days after an eastward flight	POMS, sleep diary, symptom questionnaire, KSS	Melatonin significantly improved self-rated sleep quality (p < 0.05), shortened sleep latency (p < 0.05), and reduced fatigue (p < 0.05) in subjects with jet lag. Melatonin 5 mg formulation was the most effective dosage to reduce fatigue and sleep disorders associated with jet lag after eastbound flights.	+
Petrie et al. [44]	52 participants from an Air New Zealand cabin crew (26 M/25 F) with a mean age of 34.9 ± 7.7 ^c^	overall (52/44)	5 mg melatonin capsule vs. placebo taken daily between 0700-0800, 2-3 days prior to return flight, and between 2200-0000 h until 5 days after return home^e^	VAS, SSS, retrospective jet lag VAS, POMS	Melatonin reduced the subjective effects of jet lag, reduced feelings of jet lag (p < 0.05) and led to a more rapid recovery of sleep and energy levels (p < 0.05).	+
***Insomnia (n = 4)***						
Almeida Montes et al. [45]	10 insomnia patients (6 M/4 F) with a mean age of 50 yrs. ± 12.7^c^	overall (10/10)	0.3 mg or 1 mg sustained-release melatonin capsules vs. placebo taken 60 min before bedtime, (bedtime between 2200-2300 h) for 7-day treatment period^e^	PSG, VAS, sleep diary	Melatonin did not affect sleep quality in patients with primary insomnia.	+
Wade et al. [28]Wade et al. [46]	791 participants (age/gender = ND) with primary insomnia according to the DSM-IV criteria^ac^	*treatment period:* overall (791/748), PRM (395/374), placebo (396/374); *extension period:* overall (711/555), PRM (534/421), placebo (177/134)	2 mg prolonged-release Circadin pill vs. placebo taken daily 1-2 h before bedtime (bedtime between 2100-2200 h)	National sleep foundation diary, PSQI	Melatonin (Circadin) significantly increased sleep time (p = 0.035) for individuals 18-80 years compared to placebo.	+
Garfinkel et al. [47]	34 patients (9 M/25 F) with a mean age of 68 ± 13 who were willing to discontinue current benzodiazepine therapy at some point during the study^c^	overall (34/30), CRM (18/15), placebo (16/15)	2 mg Circadin pill vs. placebo taken 2 h before bedtime (bedtime between 2100-2300 h)	Subjective sleep quality questionnaire	Melatonin significantly improved sleep quality (p = 0.04) compared to placebo, indicating that controlled-release melatonin may effectively facilitate discontinuation of benzodiazepine therapy while maintaining good sleep quality.	+
James et al. [48]	10 participants (4 M/6 F) with a diagnosis of Disorder in Initiating or Maintaining Sleep with a mean age of 33.4 ± ND^d^	overall (10/10)	1 mg or 5 mg melatonin pill vs. placebo taken 15 min before bedtime (2300 h)^e^	DSQ, VAS, SSS, EEG	Melatonin 1 mg significantly increased REM latency (p < 0.05), and produced a significant delay in REM latency after bedtime administration (p < 0.05). Melatonin 5 mg resulted in less sleep (p = 0.02) and an improvement in overall subjective sleep quality (p = 0.03) compared to 1 mg and placebo.	+
***Healthy Volunteers (n = 17)***						
***Initiation of Sleep/Sleep Efficacy (n = 7)***						
Paul et al. [49] Paul et al. [25]	23 military and civilian volunteers (9 M/ 14 F) with a mean age of 29.9 ± 10.3^c^	overall (23/ND)	6 mg time-released Circadin pill vs. placebo vs. 10 mg Zaleplon pill vs. 7.5 mg Zopiclone pill vs. 15 mg Temazepam pill taken at 0945 h on one of 5 experimental days^e^	PSG, 7 point Likert drowsiness scale	Melatonin use significantly increased sleep (p < 0.05), decreased sleep latency (p < 0.05), and increased drowsiness (p < 0.0001) immediately after psychomotor testing compared to before testing for all medications. Melatonin increased sleep and reduced sleep latency (p < 0.05) after psychomotor test sessions from 1 3/4h to 4 3/4h post-ingestion. Melatonin significantly prolonged subjective sleepiness (p < 0.001); however, the largest effects on total sleep, sleep latency and drowsiness were attributable to Zopiclone.	+
James et al. [50]	10 participants (7 M/3 F) with a mean age of 29.9 ± ND^d^	overall (10/10)	1 mg or 5 mg melatonin pill vs. placebo taken at 2245 h for one of three weeks	PSG	Melatonin 5 mg significantly prolonged REM latency (p < 0.001), suggesting that a larger dosage of melatonin may influence sleep and circadian rhythms.	+
Nave et al. [51]	6 healthy males with a mean age of 24.5 ± 0.9^c^	overall (6/6)	3 mg melatonin pill vs. placebo vs. 10 mg Flumazenil + placebo vs. 10 mg Flumazenil + 3 mg melatonin taken at 1200 h for one of four 7 h (1200-1900 h) testing periods	PSG, actigraph	Melatonin significantly decreased latency to the first appearance of sleep (p < 0.05) and increased total sleep time (p < 0.05).	+
Middleton et al. [52]	10 normal healthy male soldiers with a mean age of 23.90 ± 0.75^c^	overall (10/8)	5 mg melatonin capsule vs. placebo taken at 2000 h, 1200 or 0400 h for 15 days^e^	Sleep diaries, urine samples	Melatonin produced significant differences for sleep onset, sleep offset and activity acrophase (p < 0.001), indicating a sleep phase shift.	+
Aeschbach et al. [53]	8 volunteers (4 M/4 F) with a mean age of 27.8 ± 3.6^c^	overall (8/8)	2.1 mg melatonin patch vs. placebo patch given one hour before 8 h daytime sleep opportunity (between 0900-1700 h) on day 2 of a 36 h inpatient visit^e^	PSG, KSS, blood samples	Transdermal melatonin delivered during the daytime elevated plasma melatonin (p < 0.0001) and reduced waking (p < 0.05) after sleep onset by promoting sleep (p < 0.05) in the latter part of an 8 h sleep opportunity.	+
Attenburrow et al. [54]	15 healthy middle aged volunteers (4 M/11 F) with a mean age of 53.9 ± ND^c^	overall (15/12)	0.3 mg or 1 mg melatonin pill vs. placebo given 2h before bedtime (bedtime between 2200-2300 h) for 3 separate nights^e^	PSG, Leeds sleep evaluation questionnaire	Melatonin improved actual sleep time (p < 0.02), sleep efficiency (p < 0.02), non-REM sleep (p < 0.03) and REM sleep latency (p < 0.05) in healthy, middle-aged volunteers sleeping in their home environment.	-
Van Den Heuvel et al. [55]	10 healthy male volunteers with a mean age of 22 ± 1.1^c^	overall (10/10)	100 mg atenolol pill + 1 mg melatonin pill vs. placebo pill vs. 100 mg atenolol pill + placebo pill taken at 1900 h, 2200, 0200, or 0400 h during 3 nonconsecutive nights in the sleep laboratory^e^	MSLT, linear sleepiness rating	Melatonin did not affect sleep onset latencies and subjective sleepiness.	+
***Daytime Sleepiness (Occurrence Of)/Somnolence (n = 5)***						
Rose et al. [56]	68 participants (age/gender = ND) recruited from Santa Clara University^ac^	overall (68/53)	2 x 3 mg melatonin capsules vs. placebo taken 30 min before bedtime for 8 nights^e^	DSSEQ	Melatonin facilitated an increase in grogginess/ tiredness prior to sleep onset (p = 0.01). Additionally, the expectancy of receiving melatonin resulted in significantly higher mean ratings of grogginess/tiredness (p = 0.02).	+
Rogers et al. [57]	16 young healthy subjects (6 M/10 F) with a mean age of 21.4 ± 6^c^	overall (16/16)	5 mg melatonin capsule vs. placebo vs. 10 mg Temazepam capsule taken at 1200 h during 1 of 3 experimental sessions, each lasting from 2200 until 1900 h the following day^e^	VAS	Melatonin and Temazepam both produced a significant increase in self-reported sleepiness levels (p = 0.02) relative to placebo. However, melatonin use led to a steady increase in self-reported sleepiness levels (p = 0.006) compared to both Temazepam and placebo.	+
Krauchi et al. [58]	8 healthy male students with a mean age of 25 ± 4^c^	overall (8/8)	5 mg melatonin capsule vs. placebo taken once at 1300 h^e^	VAS, KSS, waking EEG	Melatonin administration at 1340, 1420, 1510, 1550, and 1620h increased sleepiness (p < 0.05).	+
James et al. [50]	10 participants (7 M/3 F) with a mean age of 29.9 ± ND^d^	overall (10/10)	1 mg or 5 mg melatonin pill vs. placebo taken at 2245 h daily for one week	DSQ, VAS, SSS	Melatonin did not produce differences in daily sleep or sleepiness between groups.	+
Nave et al. [51]	6 healthy males with a mean age of 24.5 ± 0.9^c^	overall (6/6)	3 mg melatonin pill vs. placebo vs. 10 mg Flumazenil + placebo vs. 10 mg Flumazenil + 3 mg melatonin taken at 1200 h for one of four 7 h (1200-1900 h) testing periods	VAS	Melatonin 3 mg significantly increased sleepiness (p < 0.02) when administered at 1200 h in the placebo + melatonin and flumazenil + melatonin conditions.	+
***Phase Shift/Hormone Changes (n = 5)***						
Bonafide et al. [59]	12 healthy volunteers (age/gender = ND)^ac^	overall (12/10)	3 mg melatonin pill + Saline (80 ml/h) IV vs. 3 mg melatonin pill + Remifentanil (0.02-0.04 ug kg) IV vs. placebo + Saline (80 ml/h) IV vs. Remifentanil (0.02-0.04 ug kg) IV + placebo, administered at 2230 h^e^	PSG, sleep diary	Melatonin did not alter normal nocturnal sleep or prevent remifentanil-induced sleep disturbance.	+
Luboshitzky et al. [60]	6 healthy males with a mean age of 23.9 ± 2.4^c^	overall (6/5)	6 mg melatonin pill vs. placebo once a day at 1700 h for 1 month	PSG	Neither melatonin nor the control influenced the majority of polysomnographic sleep parameters, however, melatonin did significantly increase REM latency (p < 0.04) and percent REM (p < 0.05) compared to baseline.	-
Gorfine et al. [61]	12 participants (2 M/10 F) with a mean age of 25 ± 4.8^c^	overall (12/ND)	2 mg melatonin drink (100 ml of 1% ethanol in water) vs. placebo drink administered every 2-3 h, starting at 1600–1700 h^e^	Bond-Lader questionnaire	Melatonin caused significant increases from predosing scores in self-reported parameters of fatigue (p < 0.001), sleepiness (p < 0.001), dreaminess (p < 0.01) and boredom (p = 0.02) and significant decreases in lucidness (p = 0.03).	-
Vandewalle et al. [62] Rajaratnam et al. [26] Rajaratnam et al. [27]	8 male subjects with a mean age of 24.4 ± 4.4^c^	overall (8/8)	1.5 mg surge-sustained-release melatonin pill vs. placebo taken at 1600 h during daily scheduled 16 h sleep opportunities for 8 consecutive days^e^	HR/HRV, blood samples, actigraph, KSS, PSG.	Melatonin successfully phase-shifted circadian rhythms (p < 0.045) without indication of deleterious effects on daytime sleepiness/mood on the day following administration.	+
Paul et al. [63]	11 normal healthy male volunteers with a mean age of 38.2 ± 9.7^ac^	overall (11/11)	3 mg sustained release melatonin capsule vs. light treatment + 3 mg melatonin capsule vs. placebo capsule vs. light treatment. Capsules were administered at 1600h on day 2, light treatment from 0600-0800h on day 3^e^	Melatonin assays, saliva samples, actigraph	Melatonin significantly increased phase advances compared to placebo condition (p < 0.0002).	+

ATS = Accumulated Time with Sleepiness scale, CRM = controlled-release melatonin, DSM-IV = Diagnostic and Statistical Manual of Mental Disorders, Fourth Edition, DSQ = Daily Sleep Questionnaire, DSSEQ = Daily Subjective Sleep Experiencing Questionnaire, EEG = Electroencephalogram, F = female, h = hour, HR = heart rate, HRV = heart rate variability, KSS = Karolinska Sleepiness Scale, M = male, mg = milligram, MSLT = Multiple sleep latency test, n = number, ND = not described, NR = not reported, POMS = Profile of Mood States, PRM = Prolonged-release circadin, PSG = Polysomnographic recording, PSQI= Pittsburgh Sleep Quality Index, REM = Rapid eye movement, SSS = Stanford Sleepiness Scale, VAS = Visual Analog Scale, wk = week ^a^power achieved, ^b^power not achieved, ^c^informed consent obtained, ^d^informed consent not obtained, ^e^crossover design.

^f^See Table 1: Quality refers to the overall SIGN 50 score, categorized as *++* (well covered; where criteria has not be filled, conclusions of the study are thought very unlikely to alter); *+* (adequately addressed; criteria that have not been adequately described are thought unlikely to alter conclusions) or *–* (poorly addressed; conclusions of study are thought likely or very likely to alter).

Note. Four [25–28] RCTs reported on different outcomes of the same study and were therefore combined; the most recent study was cited in the paper.

**Table 3:** **Grading of Recommendations Assessment, Development and Evaluation (GRADE) analysis: quality of the overall literature pool assessing melatonin for the promotion of healthy sleep patterns**

Category	Number of participants completed (Number of studies)	Confidence in estimate of Effect GRADE ^1^	Magnitude of estimate of Effect GRADE ^2^	Safety GRADE ^3^	Strength of the Recommendation ^4^
**Shift Workers**	300 (8)	C	ND	+1	None
**Jet Lag**	972 (8)	B	ND	+1	Weak, in favor
**Insomnia**	845 (4)	B	ND	+1	Weak, in favor
**Healthy Volunteers**					
*Initiation of Sleep/Sleep Efficacy*	82 (7)*	B	ND	0	Weak, in favor
*Occurrence of Daytime Sleepiness/Somnolence*	108 (5)*	B	ND	0	Weak, in favor
*Phase Shift Changes*	49 (5)	C	ND	0	None

*2 Studies [50, 51] mentioned in both groups.

There are four major domains that comprise the core of the modified GRADE methodology:

^1^Categorized as *A* (High; further research is very unlikely to change confidence in the estimate of effect); *B* (Moderate; further research is likely to have an important impact on confidence in the estimate of effect and may change the estimate)*; C* (Low; further research is very likely to have an important impact on confidence in the estimate of effect and is likely to change the estimate)or; *D* (Very Low; any estimate of effect is very uncertain).

^2^Categorized as *none* (<0.2), *small* (0.2 – 0.5), *moderate* (0.5 – 0.8), *large* (>0.8) or *not described* (ND; authors did not describe or report effect size for this review’s outcomes of interest due to the lack of author reporting).

^3^Dependent on the frequency and severity of adverse events and interactions; as *+2* (appears safe with infrequent adverse events and interactions); *+1* (appears relatively safe but with frequent but not serious adverse events and interactions); *0* (safety not well understood or conflicting); −*1* (appears to have safety concerns that include infrequent but serious adverse events and/or interactions) or; −*2* (has serious safety concerns that include frequent and serious adverse events and/or interactions).

^4^Strength of the recommendation can be determined using the following categories and criteria: *Strong recommendation in favor of or against* (very certain that benefits do, or do not, outweigh risks and burdens); *No recommendation* (no recommendations can be made) or; *Weak recommendation in favor of or against* (benefits and risks and burdens are finely balanced, or appreciable uncertainty exists about the magnitude of benefits and risks).

**Table 4:** **Reporting of dietary supplement design elements**

	Jet lag (n = 8)	Insomnia (n = 4)	Healthy volunteers (n = 15)	Shift workers (n = 8)	Total N (%)
Assessment of baseline exposure	0/8	2/4	2/15	0/8	4/35 (11%)
Control for background diet	5/8	1/4	11/15	4/8	21/35 (58%)
Description of Melatonin preparation	1/8	3/4	11/15	5/8	20/35 (57%)
Chemical analysis of Melatonin preparation	2/8	0/4	3/15	1/8	6/35 (17%)
Absorption analysis of the Melatonin preparation	1/8	2/4	9/15	1/8	13/35 (37%)
**Total N (%)**	**9/40 (22%)**	**8/20 (40%)**	**16/75 (48%)**	**11/40 (27%)**

**Table 5:** **Objective and subjective outcome measures captured in the review**

Outcome name	Shift workers	Jet lag	Insomnia	Initiation of Sleep/Sleep efficacy	Daytime sleepiness (occurrence of)/Somnolence	Phase shift/hormone changes
**Objective measures**						
Polysomnographic recording (PSG)*; Actigraph*; Saliva samples**; Blood samples**; Electroencephalogram (EEG)*; Accelerometers*; Hours of sleep*; Heart rate/Heart rate variability (HR/HRV)*; Melatonin assays*; Multiple sleep latency test(MSLT)*; Urine samples**; 5- min reaction test*	7	6	2	9	1	9
**Subjective measures**						
Visual Analog Scale (VAS)*; Sleep diaries; Stanford Sleepiness Scale (SSS)*; Profile of Mood States (POMS)*; Karolinska Sleepiness Scale (KSS)*; Daily Sleep Questionnaire (DSQ); Questionnaire; 7 point Likert Drowsiness Scale; Accumulated Time with Sleepiness Scale (ATS)*; Accumulated Time with Sleepiness Scale (ATS)*; Bond-Lader Questionnaire; Columbia Jet Lag Scale*; Daily Subjective Sleep Experiencing Questionnaire (DSSEQ); Leeds Sleep Evaluation Questionnaire*; Linear Sleepiness Rating; Pittsburgh Sleep Quality Index (PSQI)*; Retrospective ratings; Subjective Sleep Quality Questionnaire; Symptom assessments; Symptom questionnaire	21	24	8	5	6	3

*Validated Measures.

**Objective measures captured to determine melatonin bioavailability but not relevant outcomes of interest to this review.

### Overall quality assessment of individual included studies

The overall methodological quality of the RCTs was evaluated as being of the highest (++) quality, high (+) quality or poor (−) quality, according to the SIGN 50 criteria indicated in Table 1 (see Table 2 for quality scores). The majority (80%) of the 35 included RCTs were high (+) quality, with one study (3%) being of the highest (++) quality [34]. Conversely, 17.0% of studies were scored as poor quality. An appropriate and clearly focused research question was adequately addressed in 80.0% of the trials; the remaining (20.0%) addressed this area well. Over half (51.0%) of the studies had dropout rates less than 10.0% and were, therefore, considered well-covered for this criterion, whereas 31.0% of studies either did not mention dropout rates or reported rates greater than 20.0%. Regarding intention-to-treat analysis, 54.0% of the included studies were classified as poorly addressed because such analyses were not mentioned or described; 3.0% of the studies adequately addressed intention-to-treat analysis; and 43.0% addressed it well. Although the majority (86.0%) of studies poorly described their methods of allocation concealment, all (100%) studies adequately addressed blinding methods regarding treatment allocation. Methods of randomization were described poorly by 48.0% of the studies; 46.0% adequately described this process and only 6.0% did it well. Nearly all (97.0%) and the majority (55.0%) of the trials adequately addressed differences between treatment groups and baseline similarities, respectively. Results indicated that 51.0% of articles adequately covered outcome validity and reliability, whereas 37.0% covered this criterion well. Three studies [28, 29, 62] were multi-site, and one [28] poorly addressed the comparability of results among sites; the remaining two were well covered [29] or adequately addressed [62] in this area.

### Adverse events

Of the total 35 studies included in our analysis, 15 [29, 30, 33, 34, 36, 38, 39, 41–47, 52] included information on adverse events. No serious adverse events were reported. One [36] study reported that adverse events occurred, but did not describe them, and two [34, 45] reported no adverse events occurred at all. The most common adverse events were headache [29, 33, 44, 46, 47] and somnolence [33, 41, 44, 48]. Palpitations [42, 43] and abdominal pain [33, 43] were each reported in two studies. The remaining adverse events were reported infrequently, and each occurred in only one of the multiple studies: nasopharyngitis [46], arthralgia [46], tachycardia [39], dizziness [33], nausea [33], vomiting [33], nightmares [33], difficulty swallowing and breathing [38], hypnotic activity [39], heavy head [39], heartburn [43], flatulence [43], swelling of arms/legs [43], sweating/hot flash [43], exanthema [43], sleeping difficulties [44], depression [44], problems with the rectal probe [52], and sleep walking [42].

#### Effectiveness of melatonin for promoting healthy sleep outcomes

Included studies were categorized according to the intended use of melatonin in 1) shift workers and individuals with jet lag to rebalance the sleep-wake cycle; 2) persons with insomnia to promote sleep; and 3) in healthy volunteers to improve outcomes of sleep efficacy, somnolence, and/or hormonal phase shift changes (see Table 2 for full description of included studies and Table 3 for GRADE Analysis).

### Shift workers

Eight [29–36] RCTs with 300 total participants assessed the efficacy of melatonin for promoting sleep in shift workers. The majority of studies were of either high (+) [29, 30, 33, 35, 36] [or highest (++) quality [34], with two [31, 32] poor (−) quality RCTs suffering from inadequate reporting of dropout rates, concealment methods, and baseline differences between groups. Results indicated that both of the poor quality studies favored melatonin [31, 32] however, all of the high and highest quality studies were inconclusive [29, 30, 33–36] in that they favored neither melatonin nor the control. Based on the five [29, 30, 33, 34, 36] studies that reported adverse events, melatonin appears to be relatively safe, with frequent but not serious adverse events and interactions. Despite its apparent safety, and the general high quality of this literature pool, sample sizes were generally small, and results inconclusive, with no magnitude of an estimate of effect size reported. Consequently, the SMEs were not able to give any recommendation for the use of melatonin in shift workers at this time.

### Jet Lag

Eight [37–44] RCTs with 972 total participants characterized melatonin use for counteracting jet lag. Almost all of the studies were of high (+) quality [37, 39–44], with the exception of one poor (−) quality study [38], which favored neither melatonin nor control, despite a large sample size (n = 339). Of the seven high (+) quality studies, one [40] favored neither melatonin nor control. The remaining six [37, 39, 41–44] RCTs favored melatonin, including two [42, 43] large studies (n = 320 [44] and n = 160 [41]) and one [39] which noted a limitation that melatonin increased tiredness the next morning. Melatonin appears to be relatively safe based on the six [38, 39, 41–44] studies that reported adverse events, citing occasional, but not serious adverse events and interactions. Based on the high quality and favorable results reported, the SMEs concluded that in a jet lagged population, further research may have an impact on the confidence in the estimate of the effect, and as such, provide a weak recommendation in favor of melatonin use for rebalancing the sleep-wake cycle in people with jet lag.

### Insomnia

Four studies of high (+) quality [45–48] with 845 total participants assessed the efficacy of melatonin in promoting better sleep in persons with insomnia. Two [45, 48] of these studies favored neither melatonin nor control, while the remaining two [46, 47] including one large study (n = 791) [46] favored melatonin, Similar to the results in jet lag studies, limitations in sample size compromised the power to produce an effect in populations with insomnia. Despite the trend in small sample sizes and lack of effect size reporting, all four studies were high quality, showing positive effects and infrequent, non-serious adverse events; as a result, the SMEs give a weak recommendation in favor of melatonin when used to promote sleep in persons with insomnia, with the understanding that the introduction of more large, high quality studies may have an important impact on this recommendation, and potentially change the confidence in the estimate of the effect size.

### Healthy volunteers

Fifteen [28, 49–61, 63] RCTs with a total of 223 participants described melatonin use for promoting sleep in healthy volunteers. Of the 15 total studies, 12 [49–53, 55–59, 62, 63] were high (+) quality and the remaining three [54, 60, 61] were poor (−) quality. Two [54, 61]of the poor quality studies favored melatonin, whereas the third [60] favored neither melatonin nor control. Of the high quality studies, eight [49, 51–53, 56–58, 62] indicated favorable results for melatonin, although six [51–53, 57, 58, 62] had small sample sizes (n = 6 to 23; total subjects for six studies = 71). The remaining four [50, 55, 59, 63] high quality studies showed no beneficial effects for either melatonin or control groups.

Healthy volunteers were furthered subdivided into three groups based on the sleep outcome under evaluation: initiation of sleep/sleep efficacy [36, 49–51, 53–55], occurrence of daytime sleepiness/somnolence [50, 51, 56–58], and induction of phase shift/hormone changes [59–63]. Two studies [50, 51] included both initiation of sleep/sleep efficacy and occurrence of daytime sleepiness/somnolence outcomes, and were consequently included in both categories. Results for each group are described below.

#### Initiation of sleep/sleep efficacy

All except one [54] of the seven studies investigating the effect of melatonin on initiation of sleep or sleep efficacy were scored high quality, and five [49, 51–54] of them showed results in favor of melatonin. Because only one [52] study in this group reported on adverse events, citing a problem with the rectal probe, safety is not well understood. Similarly, effect sizes were not reported. Despite the lack of safety and effect size reporting and small sample sizes, however, most of the studies were high quality, reporting favorable results for melatonin use. Subsequently, the SMEs provide a weak recommendation in favor of melatonin use in a healthy population for promoting sleep.

#### Daytime sleepiness/somnolence

All five studies investigating daytime sleepiness or somnolence were high quality, and four [51, 56–58] of the five small studies favored melatonin over the control. The one study not favoring melatonin [50] was poorly powered, with a sample size of (n = 10 subjects). Because no information was reported on the frequency or severity of adverse events in any of these studies, safety is not well understood. Although this group of studies suffered from small sample sizes, methodological quality was high. As a result, the SMEs provided a weak recommendation in favor of melatonin use to improve daytime sleepiness in healthy people.

#### Phase shift/hormone changes

The five studies investigating the effects of a nighttime dose of melatonin on phase shift/ hormone changes in healthy populations were more physiologically-based with primary outcomes being a change in the biomarkers being studied, and had severe limitations in study quality compared to the other two groups. Two [60, 61] of the five studies were low quality due to methodological flaws in reporting of randomization, concealment, and dropout rates. The remaining three [59, 62, 63] studies were high quality; however, the sample sizes for all five studies were fairly low. Because neither adverse events nor effect sizes were reported in any of the studies, this information remains unknown. Given this lack of information, the SMEs could not provide any recommendation for the use of melatonin to improve hormonal phase shift changes in healthy people.

### Additional dietary supplement design elements

The authors looked at additional design elements thought to be important for understanding the specific effects related to dietary supplements (Table 4). None of the studies in shift workers or jet lagged populations reported information on baseline diet exposures, but two [46, 47] studies on insomnia and two [52, 62]in healthy populations reported this information. Four [38–40, 43] jet lag studies controlled for background diets during the study, compared to nine [49, 51, 53, 57–59, 61–63] studies in healthy volunteers, none in insomnia, and three [29, 33, 35] studies in shift workers. Several studies reported that they did not control for background diets: one jet lag [42]; one insomnia [46]; one shift worker [34], and two [52, 55] healthy volunteer studies; the remaining did not report on this information. In four [29, 40, 53, 57] studies, subjects abstained from caffeine and three [43, 58, 62] studies allowed, but limited caffeine use. Formulation of the melatonin for the intervention was described in 57% of the papers, including five [29, 30, 33, 34, 36] shift worker, one [40] jet lag, three [45–47] insomnia, and 11 [49, 50, 52, 53, 55–57, 59, 60, 62, 63] healthy volunteer studies. Melatonin supplement purity was analyzed in one [33] shift worker, two [38, 40] jet lag, no insomnia, and three [50, 53, 60] healthy volunteer studies. Finally, analysis of proper absorption of melatonin was conducted in one [35] shift worker, one [37] jet lag, two [45, 46] insomnia, and nine [45, 49, 52, 53, 55, 58–60, 63] healthy volunteer studies.

### Outcome measures

A total of 31 unique assessment tools, including 19 subjective and 12 objective measures, were utilized to measure sleep outcomes (see Table 5). Although three of the 12 objective measures were of great interest, they were not relevant outcomes of interest in this review - melatonin measurements in saliva, blood, and urine. Thirteen studies [32, 35, 36, 40, 42, 45, 48, 49, 53, 54, 58, 59, 62] used a combination of both subjective and objective assessment tools to evaluate outcomes and 85.0% of these studies received high quality scores. Twenty studies [29–31, 33, 34, 37–39, 41, 42, 44, 46, 47, 50–52, 55–57, 61], 80% of which included high quality scores, used only subjective assessment tools; four [50, 51, 60, 63] studies, three of which were high quality, used only objective assessment tools.

### Dosing as reported in the literature

The amount of melatonin provided, and frequency of administration reported in the included studies varied greatly. Oral preparations were used in amounts ranging from 0.3 to 10.0 mg/day. All except two [53, 61] studies used capsules, with 72% failing to indicate the type of capsule (e.g. hard or soft) used. Two [33, 43] studies utilized fast-release preparations in amounts ranging from 3.0 to 5.0 mg. Six [35, 45, 46, 49, 62, 63] studies utilized a sustained-release formulation in amounts ranging from 0.3 to 6.0 mg, and one [53] study utilized a patch preparation providing 2.1 mg. Only one [61] study utilized a drink preparation, where 2 mg of melatonin was provided in 100 ml of 1% ethanol in water.

## Discussion

Previous research suggests that supplementation with melatonin may help increase total sleep time in individuals suffering from sleep restriction or altered sleep schedules; relieve daytime fatigue associated with jet lag; reset the body’s sleep-wake cycle; and reduce the time it takes to fall asleep in people with delayed sleep phase syndrome [64]. In fact, a number of meta-analyses have been published to evaluate the efficacy and safety of exogenous melatonin for subjects with primary sleep disorders [65, 66], and include a range of population groups for the outcomes of sleep onset latency, total sleep duration and sleep efficiency [65] and for the prevention and treatment of jet lag [65]. The inclusion and exclusion criteria vary for each of these meta-analyses. In contrast to earlier meta-analyses, the authors of this review investigated the use of melatonin in military and civilian populations across various sleep behaviors, and divided the included literature into four distinct user groups: shift workers, individuals experiencing jet lag, persons suffering from insomnia, and healthy individuals who want to improve their sleep; although the review focused on healthy populations, the authors chose to include insomnia populations as many military personnel who have been deployed may experience some form of insomnia [67].

Unfortunately, only two studies in this review were conducted in military populations: one study evaluated melatonin for jet lag in a US Air Force Reserve Unit [40] and the other assessed melatonin for sleep efficacy in the Canadian military [25]. Importantly, both were of high quality and utilized both subjective and objective measures. The authors encourage more research in this population, but suggest that the way the authors divided the literature into user groups can be useful for making generalizations for the military exposed to disruptions in sleep behavior.

Besides the limited amount of studies available in the literature that were directly on military populations exposed to melatonin and were needed to make generalizations for this specific population, another limitation of this review is that, unlike traditional systematic reviews, the REAL process only includes RCT and systematic review study designs accessible in current English electronic databases. Although the inclusion of only English literature and exclusion of gray literature may be seen as a limitation, research has shown that doing so does not seriously compromise the implications for the majority of interventions and claims [68–71]; thus REALs and systematic reviews are usually comparable and result in the same “bottom line” conclusions about the evidence [72].

Overall, results from this review suggest that melatonin shows promise to prevent phase shifts from jet lag and improve insomnia in otherwise healthy adults, but to a limited extent; the use of melatonin in shift workers is inconclusive. According to the authors’ GRADE analysis, no recommendations could be provided in favor of melatonin with regard to promoting beneficial sleep outcomes in shift workers. More high quality studies with large sample sizes and power are needed to increase the confidence in the estimate of the effect. Although no recommendation that melatonin can improve sleep outcomes in shift workers can be made at this time, the use of melatonin in healthy adults shows potential in preventing phase shifts due to jet lag. Due to these limitations and the quality of the literature, our confidence in the estimate of the effect is moderate. A weak recommendation in favor of melatonin for use on sleep outcomes in jet lagged populations is noted.

Although the purpose of the review was to look at healthy adults, the authors also explored whether melatonin could be a viable treatment option for insomnia because the consequences of insomnia are detrimental and associated with other comorbidities. All four insomnia studies showed positive effects, despite small sample sizes; thus a weak recommendation with moderate confidence in favor of melatonin is made for individuals with insomnia for improving sleep outcomes. More studies with high quality, large sample sizes are needed to increase the confidence in the estimate of the effect.

For the studies with exclusively healthy volunteers, a weak recommendation was made in favor of melatonin use for initiating sleep or sleep efficacy, again, despite sample sizes and low power. A weak recommendation with moderate confidence was made in favor of melatonin use in healthy populations for daytime sleepiness or somnolence. In contrast, studies on phase shift/hormone changes in healthy populations were primarily of low quality with small sample sizes. As such, no conclusions can be drawn regarding melatonin’s effects on producing phase shifts or hormone changes in healthy populations at this time. Clearly more research will be needed to strengthen this information.

Whereas the majority (83.0%) of the studies included in this review were deemed as relatively good quality and limited to healthy adults, great heterogeneity existed in terms of sample sizes, assessment tools, and administration of melatonin (i.e., dose, frequency, type of preparation, timing). Although most of the assessment tools aligned with good quality studies, their lack of robustness may have been a limiting factor in achieving significant effect sizes for the outcomes. Finally, although the studies were reviewed by specific indications of use (i.e., in shift work, jet lag, insomnia, or healthy volunteers) the residual variability coupled with the above issues may have limited the conclusions that could be drawn. For example, the effects of melatonin may benefit military personnel given their training and deployment requirements, and prove to be a safe intervention to promote sleep – in both warfighters and family members with sleep issues; however, the lack of studies including military populations prevents the authors from making definitive conclusions regarding the usage of melatonin in these populations. A natural sleep aid with limited side effects would be far more advantageous than a prescription sleep drug with clearly described frequent and/or rare, unexpected side effects.

Three physiologic effects: 1) promotion of sleep onset; 2) maintenance of sleep; and 3) phase-shifting of circadia rhythms - an indirect action - and the diurnal rhythm in melatonin itself [70] have been associated with melatonin administration. Melatonin has a distinct daily secretion rhythm that is determined by the sleep-wake and light–dark cycles. Nighttime exposure to bright lights phase shifts the human circadian rhythms (core temperature, cortisol and melatonin) with a maximal effect occurring in early morning when the nadir in the body’s core temperature is achieved. Administration of melatonin has an opposite effect in that melatonin can reduce or completely block the phase shift alterations in circadian rhythms induced by bright light. [73] Physiologic administration of melatonin (i.e., 0.1 to 0.3 mg) has been shown to affect both sleep onset and maintenance qualities, whereas larger amounts (i.e., 0.5 mg) affect the phase-shifting actions of melatonin. Very small oral amounts (i.e., 0.3 mg or less) of melatonin can raise daytime plasma melatonin to night-time levels [74], which is important as acute and transient hypothermia induced by exogenously administered melatonin may be critical in the circadian phase shifting and hypnogenic actions of melatonin. Because the clinical administration of melatonin (i.e., 0.5, 3.0 and 9.0 mg doses in the daytime) has been shown to induce hypothermia in a dose-dependent manner [75], its use as a potential natural sleep therapy within the military may be limited when personnel are in cold environments, when hypothermia induced by the cold and a state of sleepiness/drowsiness could greatly affect multiple areas of performance.

As noted above, many different melatonin preparations were used throughout the included studies to include fast [33, 42] and long [35, 62] acting formulations, a melatonin patch [53] and a drink intervention [61]. These diverse preparations may have contributed to the effect size of the outcome measures. The pharmacokinetic properties of melatonin preparations can vary depending on the lipid solubility of the accompanying inert ingredients, and affect their bioavailability: 1 to 10 mg can raise plasma melatonin levels 3 to 60 times their normal peaks [74]. Importantly, exogenous melatonin undergoes extensive and rapid first-pass metabolism (approximately 30-60%) in the liver where it is first oxidized to 6-hydroxy melatonin by P450-dependent microsomal oxidases, and then largely converted to a sulfate or glucuronide derivative before being excreted into the urine or feces; about 2-3% is excreted unchanged into the urine or saliva [74]. Melatonin can be absorbed transdermally, but time to peak blood levels is delayed. Concentrations of melatonin vary across the 24-hour period with plasma levels peaking at night (~50-200 pg/ml) or a 10- to 50-fold increase from the daytime levels [76]. However, significant individual differences in peak melatonin levels after a standardized administration have been noted. In the most recent meta-analysis [66], the meta-regression technique was used to discern that higher melatonin amounts and longer duration trials were related to significantly greater effect sizes on sleep latency and total sleep time in subjects with primary sleep disorders than lower amounts. Urinary metabolites and saliva measures have also been utilized in clinical studies. The wide variety of methods reported in the literature for measuring melatonin in humans has increased the difficulty of comparing results across different studies; guidelines for the measurement and reporting of studies utilizing melatonin preparations have been recommended in order to advance the field [77].

Although no serious adverse events or health risks from melatonin use were noted in this review, potential detrimental health effects associated with using melatonin should be addressed. In healthy subjects, daytime administration of oral melatonin (0.1 to 1.0 mg) produced significant drowsiness, fatigue, and performance decrements, which appear to peak approximately three to four hours after ingestion [64, 78].

Melatonin has also been shown to reduce body temperature [10, 64, 79], which could preclude its use under conditions of cold stress. Also, use of melatonin could result in central nervous system (e.g., somnolence, headaches, increased frequency of seizures, nightmares), cardiovascular (e.g., hypotension or hypertension), gastrointestinal (e.g., diarrhea, abdominal pain), and dermatological effects [78, 79]. If melatonin is used for daytime sleep promotion, unwanted circadian phase shifts could occur; and if used to accelerate circadian phase shifts, potential unwelcome sleep promotion might occur [79]. Use of melatonin to promote daytime sleep may not be appropriate in certain military situations, and benefits and risks of use would need to be evaluated in the context of the mission.

## Conclusions

This review was conducted to critically assess the available peer-reviewed literature on the use of melatonin in military service members and in healthy subjects to determine whether melatonin might be useful in military populations. The review was limited by the inclusion of only two, though high quality, studies derived from military populations, which makes generalizations to the military less robust. Additionally, the REAL process only included RCTs published in the English language which may have limited the depth of the review. Although the majority of the studies in this review were relatively high quality and limited to healthy adults, great heterogeneity existed in terms of sample sizes, assessment tools, and the range of melatonin dosages administered.

The use of melatonin by healthy adults shows promise to prevent phase shifts from jet lag and improvements in insomnia, but to a limited extent. For the initiation of sleep and sleep efficacy, the data cannot yet confirm a positive benefit. No recommendation can be proposed for the use of melatonin in shift workers. Melatonin in a wide array of preparations and amounts demonstrates few significant and limiting adverse events. Because melatonin has a very low side effect profile and limited evidence of habituation and tolerance, its use in Service Members could be considered for specific tasks. However, additional randomized controlled trials with larger sample sizes, common assessment tools, and well-characterized interventions in physiologic dose ranges in military populations would be needed for confirmation.

## Abbreviations

REAL©: Rapid evidence assessment of the literature
NHIS: National Health Interview Survey
RCT: Randomized controlled trial
SME: Subject matter experts
PICO: Population, intervention, comparison, outcome
SIGN: Scottish Intercollegiate Guidelines Network
GRADE: Grading of Recommendation Assessment, Development and Evaluation.

## References

[1] Bliwise DL, Ansari FP (2007). Insomnia associated with valerian and melatonin usage in the 2002 National Health Interview Survey. Sleep.

[2] Peterson AL, Goodie JL, Satterfield WA, Brim WL (2008). Sleep disturbance during military deployment. Mil Med.

[3] Seelig AD, Jacobson IG, Smith B, Hooper TI, Boyko EJ, Gackstetter GD, Gehrman P, Macera CA, Smith TC (2010). Sleep patterns before, during, and after deployment to Iraq and Afghanistan. Sleep.

[4] Lentino C, Purvis D, Murphy K, Deuster P (2013). Sleep as a component of the performance triad: the importance of sleep in a military population. US Army Med Dep J.

[5] Van Camp R (2009). Zolpidem in fatigue management for surge operations of remotely piloted aircraft. Aviat Space Environ Med.

[6] Stranks E, Crowe S (2014). The acute cognitive effects of zopiclone, zolpidem, zaleplon, and eszopiclone: A systematic review and meta-analysis. J Clin Exp Neuropsychol.

[7] Paulke A, Wunder C, Toennes S (2014). Sleep self-intoxication and sleep driving as rare zolpidem-induced complex behaviour. Int J Legal Med.

[8] Wagner J, Wagner ML, Hening WA (1998). Beyond benzodiazepines: alternative pharmacologic agents for the treatment of insomnia. Ann Pharmacother.

[9] Pandi-Perumal S, Srinivasan V, Spence D, Cardinali D (2007). Role of the melatonin system in the control of sleep: therapeutic implications. CNS Drugs.

[10] Pevet P, Challet E (2011). Melatonin: both master clock output and internal time-giver in the circadian clocks network. J Physiol Paris.

[11] Dawson D, Heuvel C (1998). Integrating the actions of melatonin on human physiology. Ann Med.

[12] Krauchi K, Cajochen C, Pache M, Flammer J, Wirz-Justice A (2006). Thermoregulatory effects of melatonin in relation to sleepiness. Chronobiol Int.

[13] Zhdanova I (2005). Melatonin as a hypnotic: Pro. Sleep Med Rev.

[14] **Energy drink consumption and its association with sleep problems among U.S. service members on a combat deployment - Afghanistan, 2010** *MMWR Morb Mortal Wkly Rep* [http://www.cdc.gov/mmwr/preview/mmwrhtml/mm6144a3.htm] 23134972

[15] Capaldi VF, Guerrero ML, Killgore WD (2011). Sleep disruptions among returning combat veterans from Iraq and Afghanistan. Mil Med.

[16] Collen J, Orr N, Lettieri CJ, Carter K, Holley AB (2012). Sleep disturbances among soldiers with combat-related traumatic brain injury. Chest.

[17] Crowley SK, Wilkinson LL, Burroughs EL, Muraca ST, Wigfall LT, Louis-Nance T, Williams EM, Glover SH, Youngstedt SD (2012). Sleep during basic combat training: a qualitative study. Mil Med.

[18] Eliasson A, Kashani M, Dela Cruz G, Vernalis M (2012). Readiness and associated health behaviors and symptoms in recently deployed Army National Guard solders. Mil Med.

[19] Luxton DD, Greenburg D, Ryan J, Niven A, Wheeler G, Mysliwiec V (2011). Prevalence and impact of short sleep duration in redeployed OIF soldiers. Sleep.

[20] Macera CA, Aralis HJ, Rauh MJ, Macgregor AJ (2013). Do Sleep Problems Mediate the Relationship between Traumatic Brain Injury and Development of Mental Health Symptoms after Deployment?. Sleep.

[21] Mackowiak PA, Billings FT, Wasserman SS (2012). Sleepless vigilance: “Stonewall” Jackson and the duty hours controversy. Am J Med Sci.

[22] McGowan J, Sampson M (2005). Systematic reviews need systematic searches. J Med Libr Assoc.

[23] *SIGN 50: A guideline developer's handbook*. [http://www.sign.ac.uk/guidelines/fulltext/50/]

[24] *Grading of Recommendations Assessment, Development and Evaluation (GRADE)*. [http://www.gradingworkinggroup.org]

[25] Paul MA, Gray G, Kenny G, Pigeau RA (2003). Impact of Melatonin, Zaleplon, Zopiclone, and Temazepam on Psychomotor Performance. Aviat Space Environ Med.

[26] Rajaratnam SM, Dijk DJ, Middleton B, Stone BM, Arendt J (2003). Melatonin phase-shifts human circadian rhythms with no evidence of changes in the duration of endogenous melatonin secretion or the 24-hour production of reproductive hormones. J Clin Endocrinol Metab.

[27] Rajaratnam SM, Middleton B, Stone BM, Arendt J, Dijk DJ (2004). Melatonin advances the circadian timing of EEG sleep and directly facilitates sleep without altering its duration in extended sleep opportunities in humans. J Physiol.

[28] Wade AG, Ford I, Crawford G, McConnachie A, Nir T, Laudon M, Zisapel N (2010). Nightly treatment of primary insomnia with prolonged release melatonin for 6 months: A randomized placebo controlled trial on age and endogenous melatonin as predictors of efficacy and safety. BMC Med.

[29] Jorgensen KM, Witting MD (1998). Does exogenous melatonin improve day sleep or night alertness in emergency physicians working night shifts?. Ann Emerg Med.

[30] James M, Tremea MO, Jones JS, Krohmer JR (1998). Can melatonin improve adaptation to night shift?. Am J Emerg Med.

[31] Sadeghniiat-Haghighi K, Aminian O, Pouryaghoub G, Yazdi Z (2008). Efficacy and hypnotic effects of melatonin in shift-work nurses: Double-blind, placebo-controlled crossover trial. J Circadian Rhythms.

[32] Bjorvatn B, Stangenes K, Oyane N, Forberg K, Lowden A, Holsten F, Akerstedt T (2007). Randomized placebo-controlled field study of the effects of bright light and melatonin in adaptation to night work. Scand J Work Environ Health.

[33] Cavallo A, Ris D, Succop P, Jaskiewicz J (2005). Melatonin treatment of pediatric residents for adoption to night shift work. Ambul Pediatr.

[34] Wright SW, Lawrence LM, Wrenn KD, Haynes ML, Welch LW, Schlack HM (1998). Randomized clinical trial of melatonin after night-shift work: efficacy and neuropsychologic effects. Ann Emerg Med.

[35] Sharkey KM, Fogg LF, Eastman CI (2001). Effects of melatonin administration on daytime sleep after simulated night shift work. J Sleep Res.

[36] Jockovich M, Cosentino D, Cosentino L, Wears RL, Seaberg DC (2000). Effect of exogenous melatonin on mood and sleep efficiency in emergency medicine residents working night shifts. Acad Emerg Med.

[37] Arendt J, Aldhouse M, English J, Marks V, Arendt J, Marks M, Folkard S (1987). Some effects of jet-lag and their alleviation by melatonin. Ergonomics.

[38] Spitzer RL, Terman M, Williams JB, Terman JS, Malt UF, Singer F, Lewy AJ (1999). Jet lag: clinical features, validation of a new syndrome-specific scale, and lack of response to melatonin in a randomized, double-blind trial. Am J Psychiatry.

[39] Claustrat B, Brun J, David M, Sassolas G, Chazot G (1992). Melatonin and jet lag: confirmatory result using a simplified protocol. Biol Psychiatry.

[40] Beaumont M, Batejat D, Pierard C, Van Beers P, Denis JB, Coste O, Doireau P, Chauffard F, French J, Lagarde D (2004). Caffeine or melatonin effects on sleep and sleepiness after rapid eastward transmeridian travel. J Appl Physiol.

[41] Petrie K, Conaglen JV, Thompson L, Chamberlain K (1989). Effect of melatonin on jet lag after long haul flights. BMJ.

[42] Suhner A, Schlagenhauf P, Hofer I, Johnson R, Tschopp A, Steffen R (2001). Effectiveness and tolerability of melatonin and zolpidem for the alleviation of jet lag. Aviat Space Environ Med.

[43] Suhner A, Schlagenhauf P, Johnson R, Tschopp A, Steffen R (1998). Comparative study to determine the optimal melatonin dosage form for the alleviation of jet lag. Chronobiol Int.

[44] Petrie K, Dawson AG, Thompson L, Brook R (1993). A double-blind trial of melatonin as a treatment for jet lag in international cabin crew. Biol Psychiatry.

[45] Almeida Montes LG, Ontiveros Uribe MP, Cortes Sotres J, Heinze Martin G (2003). Treatment of primary insomnia with melatonin: a double-blind, placebo-controlled, crossover study. J Psychiatry Neurosci.

[46] Wade AG, Crawford G, Ford I, McConnachie A, Nir T, Laudon M, Zisapel N (2011). Prolonged release melatonin in the treatment of primary insomnia: evaluation of the age cut-off for short- and long-term response. Curr Med Res Opin.

[47] Garfinkel D, Zisapel N, Wainstein J, Laudon M (1999). Facilitation of benzodiazepine discontinuation by melatonin: A new clinical approach. Arch Intern Med.

[48] James SP, Sack DA, Rosenthal NE, Mendelson WB (1990). Melatonin administration in insomnia. Neuropsychopharmacology.

[49] Paul MA, Gray G, MacLellan M, Pigeau RA (2004). Sleep-inducing pharmaceuticals: a comparison of melatonin, zaleplon, zopiclone, and temazepam. Aviat Space Environ Med.

[50] James SP, Mendelson WB, Sack DA, Rosenthal NE, Wehr TE (1987). The effect of melatonin on normal sleep. Neuropsychopharmacology.

[51] Nave R, Herer P, Haimov I, Shlitner A, Lavie P (1996). Hypnotic and hypothermic effects of melatonin on daytime sleep in humans: lack of antagonism by flumazenil. Neurosci Lett.

[52] Middleton B, Arendt J, Stone BM (1997). Complex effects of melatonin on human circadian rhythms in constant dim light. J Biol Rhythms.

[53] Aeschbach D, Lockyer BJ, Dijk DJ, Lockley SW, Nuwayser ES, Nichols LD, Czeisler CA (2009). Use of transdermal melatonin delivery to improve sleep maintenance during daytime. Clin Pharmacol Ther.

[54] Attenburrow ME, Cowen PJ, Sharpley AL (1996). Low dose melatonin improves sleep in healthy middle-aged subjects. Psychopharmacology (Berl).

[55] Van Den Heuvel CJ, Reid KJ, Dawson D (1997). Effect of atenolol on nocturnal sleep and temperature in young men: reversal by pharmacological doses of melatonin. Physiol Behav.

[56] Rose DA, Kahan TL (2001). Melatonin and sleep qualities in healthy adults: pharmacological and expectancy effects. J Gen Psychol.

[57] Rogers NL, Kennaway DJ, Dawson D (2003). Neurobehavioural performance effects of daytime melatonin and temazepam administration. J Sleep Res.

[58] Krauchi K, Cajochen C, Wirz-Justice A (1997). A relationship between heat loss and sleepiness: effects of postural change and melatonin administration. J Appl Physiol.

[59] Bonafide CP, Aucutt-Walter N, Divittore N, King T, Bixler EO, Cronin AJ (2008). Remifentanil inhibits rapid eye movement sleep but not the nocturnal melatonin surge in humans. Anesthesiology.

[60] Luboshitzky R, Levi M, Shen-Orr Z, Blumenfeld Z, Herer P, Lavie P (2000). Long-term melatonin administration does not alter pituitary-gonadal hormone secretion in normal men. Hum Reprod.

[61] Gorfine T, Assaf Y, Goshen-Gottstein Y, Yeshurun Y, Zisapel N (2006). Sleep-anticipating effects of melatonin in the human brain. Neuroimage.

[62] Vandewalle G, Middleton B, Rajaratnam SM, Stone BM, Thorleifsdottir B, Arendt J, Dijk DJ (2007). Robust circadian rhythm in heart rate and its variability: influence of exogenous melatonin and photoperiod. J Sleep Res.

[63] Paul MA, Gray GW, Lieberman HR, Love RJ, Miller JC, Trouborst M, Arendt J (2011). Phase advance with separate and combined melatonin and light treatment. Psychopharmacology (Berl).

[64] Jellin J, Gregory PJ (2013). Melatonin monograph.

[65] Buscemi N, Vandermeer B, Hooton N, Pandya R, Tjosvold L, Hartling L, Baker G, Klassen TP, Vohra S (2005). The efficacy and safety of exogenous melatonin for primary sleep disorders. A meta-analysis. J Gen Intern Med.

[66] Ferracioli-Oda E, Qawasmi A, Bloch MH (2013). Meta-analysis: melatonin for the treatment of primary sleep disorders. PLoS One.

[67] Bramoweth A, Germain A (2013). Deployment-related insomnia in military personnel and veterans. Curr Psychiatry Rep.

[68] Egger M, Juni P, Bartlett C, Holenstein F, Sterne J (2003). How important are comprehensive literature searches and the assessment of trial quality in systematic reviews? Empirical study. Health Technol Assess.

[69] Hopewell S, McDonald S, Clarke M, Egger M (2007). Grey literature in meta-analyses of randomized trials of health care interventions. Cochrane Database Syst Rev.

[70] Moher D, Pham B, Klassen TP, Schulz KF, Berlin JA, Jadad AR, Liberati A (2000). What contributions do languages other than English make on the results of meta-analyses?. J Clin Epidemiol.

[71] Singh BB, Khorsan R, Vinjamury SP, Der-Martirosian C, Kizhakkeveettil A, Anderson TM (2007). Herbal treatments of asthma: a systematic review. J Asthma.

[72] Watt A, Cameron A, Sturm L, Lathlean T, Babidge W, Blamey S (2008). Rapid versus full systematic reviews: validity in clinical practice?. ANZ J Surg.

[73] Cagnacci A, Soldani R, Yen SS (1997). Contemporaneous melatonin administration modifies the circadian response to nocturnal bright light stimuli. Am J Physiol.

[74] Brzezinski A, Wurtman R, Coates P (2010). Melatonin. Encyclopedia of Dietary Supplements.

[75] Satoh K, Mishima K (2001). Hypothermic action of exogenously administered melatonin is dose-dependent in humans. Clin Neuropharmacol.

[76] Lewy AJ, Ahmed S, Jackson JML, Sack RL (1992). Melatonin shifts human circadian-rhythms according to a phase response curve. Chronobiol Int.

[77] Benloucif S, Burgess HJ, Klerman EB, Lewy AJ, Middleton B, Murphy PJ, Parry BL, Revell VL (2008). Measuring melatonin in humans. J Clin Sleep Med.

[78] *Melatonin: Prototype monograph summary*. [http://www.nap.edu/openbook.php?record_id=10882&page=367#p2000ba488960367001]

[79] *Institute of Medicine Committee on Dietary Supplement Use by Military Personnel. Use of Dietary Supplements by Military Personnel*. [http://www.ncbi.nlm.nih.gov/books/NBK3977/pdf/TOC.pdf] 20669410

